# Underdiagnosis of Fabry disease in minority ethnic groups

**DOI:** 10.1016/j.ymgmr.2025.101194

**Published:** 2025-01-17

**Authors:** Claudia L. Church Smith, Ashwin Roy, Sarah Steeds, Natalie Tuzcuoglu, Christopher Wingrove, Katherine Aitchison, Claire Radford, Laura A. Boyes, Fiona Stewart, Tarekegn Geberhiwot, Richard P. Steeds

**Affiliations:** a Queen Elizabeth Hospital, University Hospitals Birmingham NHS Foundation Trust, Birmingham, UK; b Institute of Cardiovascular Sciences, University of Birmingham, Birmingham, UK; c Amicus Therapeutics UK, Marlow, UK; d Clinical Genetics and Genomics, Birmingham Women 's and Children's NHS Foundation Trust, Birmingham, UK; e Society for Mucopolysaccharide Diseases, Amersham, UK; f Institute of Metabolism and Systems Research, University of Birmingham, Birmingham, UK

**Keywords:** Fabry disease, Genetic testing, Ethnicity, Health inequalities, Diagnosis

## Abstract

Fabry disease (FD) is a rare, pan ethnic X-linked disorder. We explored ethnic representation in our service and a national patient organisation. An audit of the University Hospitals Birmingham FD cohort revealed that 9 % of index cases are minority ethnic, compared to 18.3 % of the population of England and Wales and 51.4 % of the Birmingham population. A similar split was observed in a national patient cohort, highlighting a need to identify the reasons for under-representation.

## 1 Introduction

Fabry disease (FD) is a rare, X-linked lysosomal storage disorder, characterised by alpha-galactosidase A enzyme deficiency and the resulting multi-system accumulation of glycosphingolipids. FD is pan-ethnic and affects both sexes. Males generally exhibit more severe symptoms, while random X chromosome lyonisation results in variable phenotypes in females. Without treatment, patients may develop significant cardiac, renal, and cerebrovascular complications. Treatments aim to slow disease progression and efficacy is highest when initiated early in the course of disease [1]. The incidence rate of FD in the general population is reported around 1 in 40,000 to 1 in 100,000, however this is likely an underestimate of its true prevalence, due to the emergence of variants associated with milder and later-onset forms of FD. [2]. Underdiagnosis can be attributed to non-specific symptoms, heterogeneous presentations, and a lack of awareness of FD among clinicians. Patients can experience considerable diagnostic delays, resulting in delayed initiation of treatment [2]. Timely diagnosis can be facilitated in at-risk relatives by cascade screening [2,3].

It is well established that minority ethnic groups face numerous inequalities in accessing healthcare. This is likely due to a complex interplay of factors, including historic abuse in healthcare and research, cultural differences and sometimes language barriers. [4,5]. Lower levels of health literacy may further compound these disparities [6]. Stigma and fear of discrimination may lead individuals from minority ethnic groups to avoid seeking help from health services, leading to diagnostic delays [5]. These inequalities can result in poorer health outcomes [4,5]. Although FD is ‘pan ethnic’, and found in all demographic groups, there are no data on frequency of ethnicities with FD in the United Kingdom. The aim of this short report is to explore the representation of ethnicities across two FD patient cohorts.

## 2 Methods

We performed a retrospective audit of medical records of FD patients at the University Hospitals Birmingham (UHB), which identified 123 families. We collected the ethnicities of the index cases from their patient records. Patients self-reported their ethnicity as one of 17 ethnic data categories used in a standard format across the National Health Service. The Society for Mucopolysaccharide Diseases (MPS Society), the only registered charity in the United Kingdom that focusses on MPS diseases, the largest society of its kind, provided unpublished ethnicity data for their 510 FD members. Members self-reported their ethnicity as one of 20 ethnic categories upon registration. As the ethnic data categories differed between the UHB and MPS society cohorts, we grouped the self-reported ethnicities into 5 higher-level ethnicity categories used in the ONS census: White, South Asian, Black, Mixed, and Other ethnic backgrounds. For example, NHS coding allows selection of White – British, White – Irish, White – any other White background, and patients selecting any of these categories were grouped as white, whereas selection of Asian or Asian British - Indian, Asian or Asian British – Pakistani, Asian or Asian British - Bangladeshi, or Asian or Asian British – any other Asian background were grouped as Asian.

## 3 Results

Within the UHB cohort, 111 index cases (90 %) self-described their ethnicity as White, 5 (4 %) as South Asian, 1 (1 %) as Black, 2 (2 %) as Mixed, and 3 (2 %) as belonging to other ethnic groups. One (1 %) index case did not disclose their ethnicity. Within the MPS Society FD cohort, 416 (81.5 %) members provided their ethnicity. Of these, 387 (93 %) identified as White, 17 (4.1 %) identified as South Asian, 3 (0.7 %) as Black, 4 (1 %) as Mixed and 5 (1.2 %) as Other ethnic backgrounds.

## 4 Discussion

**Fig. 1:**
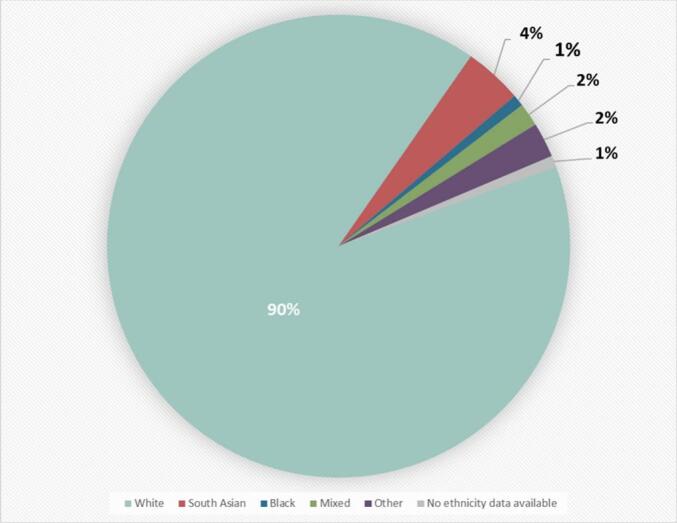
Pie chart illustrating the split of ethnicities across the UHB cohort.

Birmingham is one of two super-diverse cities in the UK, where citizens from minority ethnic backgrounds make up over half of the total population. Despite this, we identified in the UHB cohort that only 9 % of index cases were classified as minority ethnic (Fig. 1), dropping to 7 % in the MPS cohort. As FD is pan-ethnic, the ethnicities of patients in FD cohorts would be expected to resemble the ethnic diversity of the local population. Across England and Wales, 18.3 % of the population are minority ethnic. This rises to 23 % in the West Midlands, where most of the UHB cohort reside, and to 51.4 % in Birmingham [7]. The results demonstrate a lack of diversity within both cohorts, which suggests FD is potentially underdiagnosed in minority ethnic groups.

A possible explanation for this finding is that the common and well characterised variants seen in white populations are not seen in minority ethnic populations. The limited availability of information about the clinical manifestations of FD in minority ethnic populations may complicate the interpretation of any identified genetic variants. This is a wider issue in genetics and rare disease, as those from minority ethnic populations are not adequately represented in genomic datasets [8]. Furthermore, with respect to the cohort from the MPS Society, there is the potential for bias in these data due to the possibility that certain ethnic communities may be more reluctant to join patient organisations and registries.

Underdiagnosis poses a risk to patients, therefore overcoming barriers to diagnosis in minority ethnic groups is necessary to promote more favourable health outcomes. Increasing awareness of FD among healthcare professionals may increase diagnostic rates. Access to primary care is seen as more equitable for minority ethnic populations [5], thus increasing awareness of FD among primary care clinicians may facilitate diagnosis within these groups. Further research into clinical manifestations of FD in diverse groups may help clarify the pathogenicity of variants, resulting in increased diagnoses in these groups whilst also increasing confidence in health services [8].

The availability of interpreters can help overcome language barriers and cultural competence training for healthcare professionals can improve patient experience, resulting in more individuals from minority ethnic backgrounds seeking help from health services [4,5]. Learning from new roles such as community engagement experts and Core20PLUS5 connectors already working in the system to address health inequalities more broadly will be fundamental in engaging rare disease patients from underrepresented communities and supporting their journey to diagnosis.

## 5 Conclusion

There are low numbers of minority ethnic patients with FD in a major regional clinical service and national patient representative populations compared to white patients. Further research should be performed to identify why this may be the case and to ensure that any barriers to the diagnosis of FD in minority ethnic groups can be identified and overcome.

## CRediT authorship contribution statement

**Claudia L. Church Smith:** Writing – review & editing, Writing – original draft, Methodology, Investigation, Formal analysis, Data curation, Conceptualization. **Ashwin Roy:** Writing – review & editing, Writing – original draft, Supervision, Conceptualization. **Sarah Steeds:** Writing – review & editing, Investigation, Conceptualization. **Natalie Tuzcuoglu:** Writing – review & editing, Project administration, Funding acquisition, Conceptualization. **Christopher Wingrove:** Writing – review & editing, Conceptualization. **Katherine Aitchison:** Writing – review & editing, Conceptualization. **Claire Radford:** Writing – review & editing, Conceptualization. **Laura A. Boyes:** Writing – review & editing, Methodology, Conceptualization. **Fiona Stewart:** Writing – review & editing, Supervision, Resources, Conceptualization. **Tarekegn Geberhiwot:** Writing – review & editing, Conceptualization. **Richard P. Steeds:** Writing – review & editing, Writing – original draft, Supervision, Resources, Methodology, Investigation, Conceptualization.

## Declaration of competing interest

The authors declare the following financial interests/personal relationships which may be considered as potential competing interests.

Claudia Church Smith's role is funded as part of a collaborative working agreement between 10.13039/501100010745UHB and 10.13039/100015362Amicus Therapeutics.

Natalie Tuzcuoglu and Chris Wingrove are employed by Amicus Therapeutics UK.

## Data Availability

The data that has been used is confidential.
